# Regulatory Roles of Adiponectin in Animal Reproduction: Molecular Insights from the Hypothalamic–Pituitary–Gonadal Axis

**DOI:** 10.3390/biom16060832

**Published:** 2026-06-04

**Authors:** Yixiang Tian, Xing Wu, Lujie Zhang, Yujie Gong, Xiangtao Kang, Yadong Tian, Yulong Guo

**Affiliations:** 1 College of Animal Science and Technology, Henan Agricultural University, Zhengzhou 450046, China; t13071027821@163.com (Y.T.); wuxing6871@163.com (X.W.); zlj18236117037@163.com (L.Z.); gongyj1118@163.com (Y.G.); xtkang2001@263.net (X.K.); 2 Henan Key Laboratory for Innovation and Utilization of Chicken Germplasm Resources, Zhengzhou 450046, China

**Keywords:** adiponectin, AdipoR1/2, hypothalamic–pituitary–gonadal axis, reproduction, regulation

## Abstract

Adiponectin, an adipocyte-derived adipokine, plays a key regulatory role in physiological processes such as energy balance, glucose metabolism, and fatty acid oxidation. Evidence from animal studies indicates that adiponectin is involved in the regulation of reproductive performance by mediating molecular crosstalk between energy metabolism and the reproductive system. As the core regulatory center for animal reproduction, the hypothalamic–pituitary–gonadal (HPG) axis exhibits a close link between its functional state and the body’s energy homeostasis. This review systematically summarizes the expression patterns and molecular mechanisms of adiponectin and its receptors at different levels of the animal HPG axis. It provides a theoretical basis for future research and applications of adiponectin in animal reproduction.

## 1. Introduction

**Figure 1:**
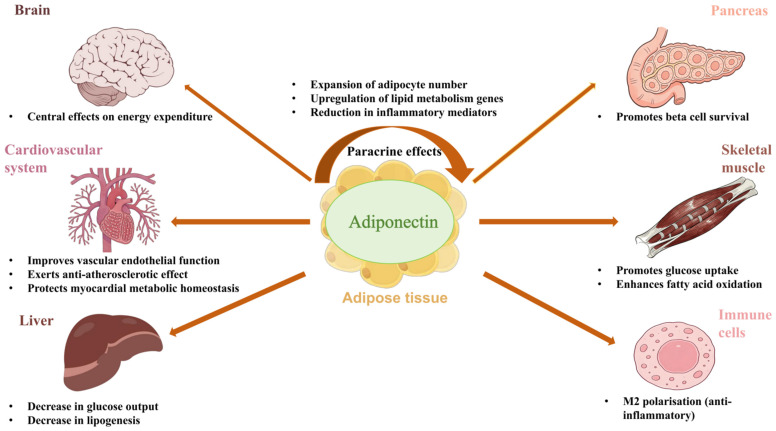
Metabolic effects of adiponectin.

Adipose tissue is increasingly regarded not only as an energy storage site but also as an endocrine organ that contributes to the regulation of systemic homeostasis. Through the secretion of a diverse set of bioactive molecules, collectively termed adipokines, it conveys information about nutritional and metabolic status to multiple physiological systems [1,2,3]. Among these factors, adiponectin is one of the most extensively studied. Since its discovery, it has been consistently linked to central aspects of metabolic regulation, including energy homeostasis, glucose metabolism, insulin responsiveness, and fatty acid oxidation. These metabolic processes are closely related to animal reproduction, because adequate energy availability, glucose metabolism, and lipid utilization are required for GnRH secretion, gonadotropin release, gonadal steroidogenesis, follicular development, gametogenesis, and early embryonic growth. Collectively, these actions support stable metabolic function at the whole-organism level [4,5,6,7] (Figure 1).

Beyond its established metabolic functions, adiponectin is increasingly implicated in the regulation of reproductive processes. Evidence from multiple species indicates that circulating adiponectin and its signaling pathways are associated with variation in reproductive traits and outcomes, consistent with a role in linking energy status to reproductive function [8,9,10,11]. Reproductive function in mammals and birds is coordinated by the hypothalamic–pituitary–gonadal (HPG) axis, whose output is tightly coupled to energetic sufficiency. Under negative energy balance, such as fasting or sustained high energy expenditure, HPG axis activity is commonly attenuated, prioritising survival over reproduction [12]. In this context, adiponectin, which is closely associated with adiposity and metabolic state, acts as a potential upstream regulator that can modulate reproductive endocrine signaling at multiple levels of the HPG axis [13,14].

Clarifying how adiponectin is integrated into HPG axis regulation will refine current understanding of the endocrine networks that coordinate metabolism and reproduction, and may also provide insight into the mechanisms underlying metabolic disorder-associated reproductive dysfunction, including polycystic ovary syndrome (PCOS). Despite the growing body of evidence, a comprehensive synthesis of the systemic roles of adiponectin and its receptors in animal reproduction has yet to be fully established. Here, we summarize current knowledge of their expression patterns, principal mechanisms of action, and recent advances, with particular emphasis on their functions within the HPG axis, in order to provide a clearer basis for future investigation.

## 2. The Structure and Function of Adiponectin and Its Receptors

Adiponectin is an adipocyte-derived cytokine predominantly produced by adipose tissue. It was first identified in 1995 by the Scherer group, who named it Acrp30 in 3T3-L1 adipocytes through subtractive cDNA library screening [15]. Following this discovery, several research teams confirmed the existence of this cytokine using a variety of techniques, such as differential mRNA display, cDNA library screening, and plasma protein purification. These studies led to the assignment of several alternative names, including AdipoQ, APM1, and GBP28 [16,17,18]. It was not until 1999 that the Matsuzawa group formally introduced the unified term “adiponectin” [19], which has since become widely recognized in the scientific community.

**Figure 2:**
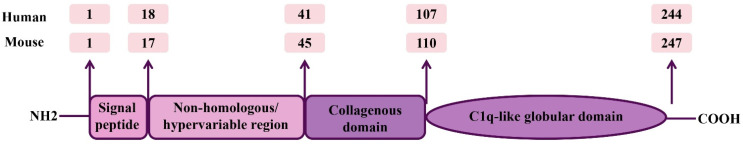
Domain Structure of Human and Mouse Adiponectin. Different colors are used to distinguish the structural domains of adiponectin. Numbers indicate the amino acid positions corresponding to the domain boundaries in human and mouse adiponectin proteins.

The human *AdipoQ* gene is located on chromosome 3 and spans approximately 15.8 kb, consisting of three exons and two introns [18]. It encodes a 244-amino-acid precursor protein, whereas the murine ortholog consists of 247 amino acids and shares approximately 83% sequence identity with the human protein (Figure 2). A key feature of adiponectin biology is that its functional diversity arises largely from post-translational modification. After hydroxylation and glycosylation in the endoplasmic reticulum, adiponectin assembles through stepwise oligomerization into three major circulating complexes: low-molecular-weight trimers (LMW, ~90 kDa), medium-molecular-weight hexamers (MMW, ~180 kDa), and high-molecular-weight multimers (HMW, ~360–540 kDa) [20,21]. In vivo, adiponectin is synthesized in adipocytes but acts largely as oligomeric species after secretion, and these multimers are increasingly viewed as functionally non-equivalent. In particular, HMW adiponectin is frequently considered the most metabolically active circulating fraction and has been reported to correlate positively with whole-body insulin sensitivity, whereas obesity and metabolic syndrome are commonly associated with reductions in both HMW abundance and total adiponectin levels [22]. By contrast, globular adiponectin (gAd) is present at relatively low concentrations in circulation but exhibits higher affinity for certain receptors than full-length adiponectin (fAd). This has led to the view that gAd may preferentially act in local or paracrine contexts, contributing to the regulation of inflammatory microenvironments and tissue-specific metabolic processes [23,24,25,26]. These features highlight that adiponectin should be considered a group of structurally and functionally distinct isoforms rather than a single uniform hormone, and that isoform composition is an important variable when comparing findings across studies.

Adiponectin exerts its biological effects through three receptor classes, among which AdipoR1 and AdipoR2 are the principal signalling receptors. Both display an atypical seven-transmembrane topology, with an intracellular N-terminus and an extracellular C-terminus, in contrast to classical G protein-coupled receptors [27]. A third binding partner, T-cadherin (CDH13), is highly expressed in vascular endothelial cells and cardiomyocytes. Although it does not directly initiate canonical intracellular signalling, it preferentially binds multimeric adiponectin, particularly HMW forms, and is thought to influence adiponectin distribution and tissue retention [28,29,30]. Differences in receptor distribution and ligand preference contribute to the tissue specificity of adiponectin signalling. AdipoR1, which is highly expressed in skeletal muscle, shows relatively high affinity for gAd and is commonly associated with AMPK and p38 MAPK pathways that promote glucose utilisation and fatty acid oxidation. In contrast, AdipoR2 is enriched in the liver, displays a preference for fAd, and is linked to PPARα-mediated pathways involved in lipid metabolism and gluconeogenesis [31,32,33,34,35]. This receptor-level organisation provides a basis for understanding the tissue-specific effects of adiponectin and may also help explain how its signalling extends from peripheral metabolic regulation to neuroendocrine control, as the biological outcome depends not only on circulating levels but also on isoform composition and receptor context.

## 3. The Regulatory Role of Adiponectin and Its Receptors in Animal Hypothalamus

The hypothalamus sits at the top of the reproductive neuroendocrine hierarchy. By integrating internal energy signals with external and physiological cues, it governs reproductive output largely through the release of neuropeptides, most prominently gonadotropin-releasing hormone (GnRH) and gonadotropin-inhibiting hormone (GnIH). In this context, the discovery that adiponectin signalling components are present in hypothalamic tissue has provided a plausible molecular route through which metabolic state can be translated into reproductive endocrine responses. Transcript-level analyses have detected adiponectin and adiponectin receptor mRNAs (*AdipoR1* and *AdipoR2*) in the hypothalamus of humans, rodents, and poultry [36,37,38,39,40,41]. Complementary immunohistochemical and molecular approaches further support a broad hypothalamic distribution of adiponectin and its receptors across vertebrates, including humans, mice, pigs, and chickens, suggesting that hypothalamic adiponectin signalling is a conserved feature rather than a lineage-restricted novelty. Human anatomical data provide useful anchoring points for this receptor map. Psilopanagioti et al. reported strong AdipoR1 immunoreactivity in neurons of the lateral hypothalamic zone (LHZ), a region comparable to the lateral hypothalamic area (LHA) described in avian neuroanatomy, and in the Meynert basal nucleus (NBM) [42]. In rodents (mice and rats), both AdipoR1 and AdipoR2 have been localised within the hypothalamus, with AdipoR2-immunopositive cells reported to be less abundant than AdipoR1-immunopositive cells [43,44]. In chickens, expression signals for adiponectin and its receptors have been detected in several nuclei relevant to metabolic sensing and reproductive control, including the paraventricular nucleus (PVN), LHA, dorsomedial nucleus (DMN), medial mammillary nucleus (MM), and the infundibular nucleus (IN) [45]. Beyond presence and distribution, an especially informative observation is that adiponectin receptors can colocalise with reproduction-related neuropeptide systems, including GnRH-associated neuronal populations [43,45]. This colocalisation does not by itself establish directionality of regulation, but it does provide a direct anatomical substrate for adiponectin to influence upstream steps of the HPG axis.

Mechanistic studies have begun to outline how adiponectin signalling could operate within hypothalamic circuits. In mammals, adiponectin binding to AdipoR1 has been linked to activation of the AMP-activated protein kinase (AMPK) pathway, which acts as a cellular energy sensor and coordinates energy homeostasis through phosphorylation of downstream substrates [46,47]. Adiponectin signalling through AdipoR2 has also been connected to rapid changes in GnRH neuron physiology. Klenke et al. showed that adiponectin can influence GnRH neuronal membrane potential and calcium dynamics through a PKCζ/LKB1/AMPK cascade, providing a mechanistic explanation for how adiponectin may suppress GnRH neuronal activity at the cellular level [43]. In parallel, adiponectin can act through transcriptional routes. Wen et al. reported that adiponectin suppresses hypothalamic KISS1 promoter activity through coordinated actions of AMPK and specificity protein 1 (SP-1), thereby indirectly reducing GnRH synthesis and secretion and forming an “adiponectin–KISS1–GnRH” regulatory cascade [48,49]. These studies collectively converge on a coherent functional theme: adiponectin signalling can tune the hypothalamic gatekeeper role of GnRH, thereby influencing the overall endocrine tone of reproduction.

Evidence from avian systems reinforces the centrality of GnRH regulation while also highlighting potential taxon-specific wiring. In chickens, AdipoQ has been reported to inhibit GnRH release through AMPK- and phosphoinositide 3-kinase (PI3K)-linked pathways [50]. When viewed together with mammalian work, a recurring conclusion emerges that adiponectin targets hypothalamic control of GnRH secretion as a key node for reproductive regulation. At the same time, an important interspecies divergence needs to be handled carefully in reviews. In mammals, a substantial portion of the inhibitory influence of adiponectin on GnRH output is frequently discussed in relation to KISS1-mediated signalling. In birds, however, the canonical kisspeptin pathway is not always conserved in the same genomic form across lineages, and chickens have been described in some comparative analyses as lacking an orthologous KISS1 sequence. This raises the likelihood that birds rely on alternative upstream regulators, with GnIH being a biologically plausible candidate, although the molecular architecture and circuit-level logic of such alternative pathways remain incompletely resolved. Clarifying these differences is not a minor taxonomic detail; it directly affects how mammalian models are extrapolated to poultry and other avian species in both basic and applied reproductive biology.

From a metabolic perspective, these findings suggest that adiponectin may participate in the communication between energy status and reproductive endocrine regulation. Adiponectin is closely associated with glucose utilization, lipid metabolism, fatty acid oxidation, and insulin sensitivity [6,7,25], and several hypothalamic studies have linked its actions to AMPK-dependent energy-sensing pathways [43,50]. The reported effects of adiponectin on GnRH neuronal activity may therefore be related to metabolic signaling within hypothalamic reproductive circuits, although this possibility requires further validation. In obesity or overnutrition, circulating adiponectin is generally reduced [4,19,25], indicating that obesity-associated reproductive dysfunction cannot be explained simply by increased adiponectin-mediated GnRH inhibition. Instead, altered adiponectin signaling may represent one component of the broader metabolic and inflammatory disturbance associated with impaired HPG axis regulation [9,10]. Under undernutrition or negative energy balance, reproductive activity is often suppressed through reduced hypothalamic GnRH drive; however, whether adiponectin directly contributes to this process in livestock and poultry remains unclear [13,14,38,41].

## 4. Regulatory Effects of Adiponectin and Its Receptors on Animal Pituitary

The pituitary gland acts as the principal endocrine relay between hypothalamic neuropeptide signals and gonadal function. By synthesising and secreting the gonadotropins luteinizing hormone (LH) and follicle-stimulating hormone (FSH), it directly regulates core reproductive events, including follicular growth, ovulation, corpus luteum formation, and gonadal steroid secretion. Given this pivotal role, it is not surprising that adiponectin signalling components have been detected in pituitary tissue across multiple taxa. Studies have reported adiponectin and its receptors at the mRNA and protein levels in pituitaries from mice, rats, cattle, humans, and chickens [42,51,52,53,54]. Immunofluorescence-based colocalisation analyses further suggest that adiponectin and its receptors are enriched in the anterior pituitary [45], which is consistent with potential actions on endocrine cell types responsible for gonadotropin and prolactin secretion.

Functional evidence indicates that pituitary adiponectin signalling is sensitive to physiological context, particularly reproductive stage and species. In porcine pituitary tissue, Kiezun et al. documented estrous cycle-dependent variation in AdipoR1 and AdipoR2 expression, supporting a model in which adiponectin may regulate pituitary activity through autocrine or paracrine mechanisms that couple metabolic state to reproductive output [52]. In vitro exposure of pituitary preparations to recombinant adiponectin has been reported to produce dose- and time-dependent effects, and neuroendocrine experiments have suggested that adiponectin can influence GnRH- and GnIH-related regulation in a manner that depends on both tissue type and experimental timing [55]. This observation may be partly explained by the direct action of adiponectin on pituitary cells. Previous studies have shown that adiponectin can activate AMPK signaling in gonadotrope cells and modulate GnRH receptor expression as well as LH and FSH synthesis or secretion [56,57]. This indicates that pituitary adiponectin signaling may couple metabolic sensing to gonadotropin output through AMPK-related pathways, although whether this involves direct changes in mitochondrial oxidative metabolism in gonadotrophs remains to be clarified. Notably, porcine pituitary adiponectin expression also exhibits estrous cycle-dependent phase specificity; in vitro treatment of porcine pituitary cells with adiponectin enhances FSH release without affecting LH secretion. In contrast, adiponectin exposure reduces LH secretion in cultured rodent pituitary cells [58]. Furthermore, GnRH treatment has been shown to downregulate adiponectin expression in rat pituitary cells [59]. Li et al. reported that adiponectin may inhibit GnRH secretion in sows through AdipoR1-mediated signaling [60]. It is hypothesized that adiponectin exerts inhibitory effects on pituitary LH and FSH secretion through AdipoR1 and AdipoR2, either via the GnRH receptor or through direct actions on gonadotrophs—a notion supported by findings from Wu et al. [56] and Lu et al. [57]. Notably, however, in primary pituitary cells isolated from baboons and macaques, adiponectin had no significant effect on LH or FSH secretion [61]. Instead, this study revealed that adiponectin specifically promotes prolactin (PRL) secretion via the adenylyl cyclase (AC)/protein kinase A (PKA), PI3K, and calcium signaling pathways [61]. In contrast, adiponectin suppresses PRL secretion in chickens [57].

## 5. Regulatory Roles of Adiponectin and Its Receptors in Animal Gonads

### 5.1. Regulatory Roles of Adiponectin and Its Receptors in the Animal Ovary

**Figure 3:**
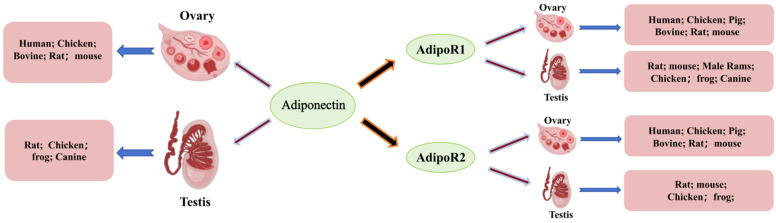
Expression of Adiponectin and Its Receptors in Reproductive Tissues of Various Animal Species. Different arrow colors were used only for visual distinction and do not indicate different biological relationships.

The ovaries serve as the core executive organs for reproductive function in female animals, orchestrating critical reproductive processes including follicular development, oocyte maturation, ovulation, corpus luteum formation, and estrogen/progesterone secretion. Their functional homeostasis directly determines the reproductive potential of female animals. Extensive studies have demonstrated that adiponectin and its receptors (AdipoR1 and AdipoR2) are expressed in ovarian tissues across multiple animal species, including humans [62,63], rats [64,65], mice [66], cattle [67], pigs [68], and chickens [69] (see Figure 3), which exhibit significant cell-type specificity. AdipoR1 and AdipoR2 are widely distributed across species in oocytes, granulosa cells, theca cells, and luteal cells [70,71]. Notably, adiponectin expression exhibits cell-type-specific differences. For instance, in certain species, the mRNA levels of adiponectin are significantly higher in theca cells than in granulosa cells [65,72]. Furthermore, expression levels of the adiponectin system are not static but undergo dynamic changes in response to follicular developmental stages and ovarian physiological status. Li et al. reported that AdipoQ and AdipoR2 expression increases with follicular volume, reaching peak levels in F1 follicles and the lowest levels in F5 follicles [73]. In bovine ovaries, mRNA expression of AdipoR1 and AdipoR2 is significantly upregulated during terminal follicular growth and the active luteal phase [67], supporting their potential involvement in follicular dominance selection and corpus luteum maintenance. However, Chabrolle et al. observed divergent expression patterns during chicken follicular development: adiponectin expression in granulosa cells declines with follicular maturation, while its expression in theca cells increases [69]. This dynamic expression pattern indicates that the adiponectin system may perform distinct regulatory functions at different stages of follicular development, providing key evidence for the direct regulatory role of adiponectin in hen ovarian function. While traditional molecular biological approaches have uncovered these regulatory patterns, there remains a scarcity of high-resolution single-cell RNA sequencing (scRNA-seq) data to precisely delineate the expression landscapes of adiponectin, AdipoR1, and AdipoR2 across distinct ovarian cell subpopulations and along continuous developmental trajectories.

Steroid hormone synthesis is one of the core functions of the ovary, being pivotal for follicular development, ovulation, and systemic endocrine homeostasis. Adiponectin can directly modulate this complex process via its receptors; however, its mode of action and underlying mechanisms remain remarkably complex. Chabrolle et al. have reported that physiological doses of recombinant human adiponectin (5 or 10 μg/mL) enhanced steroid hormone secretion in IGF-1-stimulated cells [63,65]. This effect is mediated by potentiated IGF-1 receptor signaling in rats and upregulated expression of CYP19A1—a key enzyme in estrogen biosynthesis—in humans [63,65]. In the fruit bat (Cynopterus sphinx), in vivo administration of adiponectin increased circulating progesterone (P_4_) and estradiol (E_2_) levels while concurrently upregulating ovarian AdipoR1 expression [74]. Complementary in vitro experiments confirmed that adiponectin can upregulate the expression of luteinizing hormone receptor (LHR), steroidogenic acute regulatory protein (StAR), and 3β-hydroxysteroid dehydrogenase (3β-HSD), thereby promoting progesterone synthesis. In geese, in vitro treatment of granulosa cells with 2.5 μg/mL recombinant adiponectin for 24 h upregulated StAR and CYP11A1 mRNA and protein expression, along with a significant enhancement in P_4_ secretion [75]. Similar stimulatory effects have been observed in rats [76], cattle [77,78], and humans [65], supporting a conserved regulatory role of adiponectin in steroidogenesis across phylogenetically diverse species. Concurrently, other studies have documented inhibitory effects of adiponectin. For instance, AdipoRon—a synthetic adiponectin agonist—was found to suppress estradiol E_2_ production and cell proliferation in human luteinized granulosa cells [79]. This inhibitory effect may represent a negative feedback mechanism that serves to prevent excessive hormone synthesis and secretion. Intriguingly, in laying hens, Li et al. [73] reported that AdipoQ and its receptors were expressed in growing ovarian follicles, including prehierarchical follicles and preovulatory hierarchical follicles, with F1–F5 referring to the first to fifth largest preovulatory follicles in the follicular hierarchy. They further observed that AdipoR1 showed higher expression than AdipoR2 in follicles/granulosa cells, and that treatment with 5 and 10 µg/mL AdipoRon significantly stimulated P_4_ secretion but inhibited E_2_ secretion. Whether these species-specific regulatory discrepancies are attributed to the unique reproductive and ovulatory characteristics of poultry warrants further investigation.

Adiponectin and its receptors are extensively involved in core reproductive processes, including oocyte maturation, follicular development, and early embryonic development [66,80,81]. In humans, accumulating evidence indicates that adiponectin, AdipoR1, and AdipoR2 are closely associated with early embryonic development in patients with PCOS. Specifically, AdipoR2 expression is significantly upregulated in cumulus cells associated with oocytes exhibiting blastocyst developmental competence, while AdipoR1 exhibits a similar high-expression pattern in obese PCOS patients [82]. In C. sphinx, pre-hibernation accumulation of white adipose tissue induces a decline in circulating adiponectin levels, which is associated with downregulated ovarian expression of AdipoR1, insulin receptors (IR), and glucose transporters (GLUTs). This cascade of events sequentially reduces ovarian glucose uptake, impairs progesterone synthesis, and ultimately results in embryonic diapause [83]. The fruit bat model therefore provides a concrete example in which adiposity, adiponectin signaling, ovarian glucose metabolism, steroidogenesis, and reproductive arrest occur within the same physiological context, suggesting a possible connection between adiponectin signaling and cellular energy metabolism in ovarian function, although direct causality remains to be established [83]. In ovarian follicles, coordinated glucose utilization, lipid metabolism, mitochondrial activity, and oxidative phosphorylation are required to support steroidogenic enzyme activity, oocyte maturation, follicular growth, and early embryonic development [53,70,73]. Future studies should further examine whether changes in AMPK activity, glucose transporter expression, fatty acid oxidation, mitochondrial function, and OXPHOS-related gene expression are involved in adiponectin-associated alterations in progesterone/estradiol secretion, oocyte competence, and embryo development. In chickens, adiponectin concentrations in amniotic fluid show a strong positive correlation with embryonic body weight [84], and the hormone has been validated to promote follicular growth and follicular hierarchy establishment [85]. In addition to adiponectin itself, its receptor agonist, AdipoRon, also facilitates the initiation of oocyte maturation in mice, increasing the incidence of this process by 12% [66]. Additionally, Mohan et al. [86] employed adiponectin knockout (KO) mouse models and demonstrated that adiponectin deficiency does not affect fetal growth, but induces placental dysfunction and elevates fetal triglyceride (TG) burden. In goats, Adiponectin at physiological concentrations (5–10 μg/mL) significantly reduces the proportion of oocytes with uninitiated maturation, promotes oocyte progression to metaphase I (MI) and metaphase II (MII) of meiosis, and facilitates the steady transition of oocytes from an immature to a fully mature state [87]. This positive regulatory effect is mediated through activation of the MAPK MEK 1/2 signaling pathway [88]. In swine, 30 μg/mL recombinant adiponectin accelerates oocyte meiotic maturation, while 15 μg/mL adiponectin effectively enhances embryo cleavage rates, blastocyst formation rates, and total blastocyst cell numbers (TCN). It also improves the developmental potential of cloned embryos by alleviating endoplasmic reticulum stress and suppressing apoptotic signaling pathways [89,90]. In poultry, adiponectin not only promotes follicular growth and hierarchical development in chickens but also its receptor agonist, AdipoRon, facilitates reproductive hormone secretion and gonadal development via the HPG axis [85]. In contrast, in cattle, 5 or 10 μg/mL adiponectin has no significant effect on oocyte meiotic maturation [70,91]. This interspecies discrepancy in adiponectin’s regulation of oocyte maturation and reproductive processes emphasizes the species-specificity of its biological functions, which may be attributed to inherent differences in reproductive physiology (e.g., ovulatory patterns, follicular development dynamics) and adiponectin signaling pathway components across species. This unresolved question demands further in-depth investigation to elucidate the underlying molecular and physiological mechanisms.

### 5.2. Regulatory Roles of Adiponectin and Its Receptors in the Animal Testis

The testes are essential organs in male reproductive function, carrying out vital processes such as spermatogenesis, sperm maturation, and testosterone secretion. Maintaining homeostasis within the testes is crucial for reproductive health and relies heavily on the integrated coordination of metabolic and reproductive signaling pathways. Numerous studies have documented the expression of adiponectin and its receptors, AdipoR1 and AdipoR2, across a range of testicular tissues from various species, including mice [91], rats [92], rams [93], frogs [94], canines [95], and chickens [85,96] (Figure 3), with clear distinctions in their cell-type specificity and dependence on developmental stage. In mice, AdipoR1 is predominantly localized in Leydig cells, while AdipoR2 is mainly found in spermatids within the seminiferous tubules [97]. Similarly, in bull sperm, adiponectin shows a marked enrichment in the caudal region, AdipoR1 is concentrated in the sperm membrane, particularly in the equatorial and acrosomal regions, and AdipoR2 is primarily located at the sperm head, with a prominent band at the equatorial line [98]. Additionally, in Iranian fat-tailed rams, mRNA expression of AdipoQ, AdipoR1, and AdipoR2 has been detected not only in the testes but also in various other male reproductive tract tissues, such as the caput, corpus, and cauda epididymis. Notably, expression levels of these genes are significantly higher in the epididymal corpus compared to the caput and cauda regions [99]. In chickens, AdipoQ and AdipoR1 are expressed in peritubular cells surrounding seminiferous tubules and testicular Leydig cells, while AdipoR2 is localized to Leydig cells and the lumen of seminiferous tubules [100]. From a developmental perspective, the expression levels of adiponectin and its receptors gradually increase from the prenatal stage, peak during puberty, and persist at elevated levels into the aging stage [101]. Notably, the mRNA levels of AdipoR1 and AdipoR2 in adult chicken testes are 8.3-fold and 9-fold higher, respectively, than those in juvenile chickens [100], highlighting their pivotal role in testicular sexual maturation and functional establishment.

Notably, the testes are not only target organs for adiponectin but also endogenously capable of synthesizing and secreting this adipokine. Furthermore, the concentration of adiponectin in seminal plasma exhibits a positive correlation with that in plasma [102,103], which further supports its local regulatory role in reproductive processes. Adiponectin modulates testicular function by activating multiple downstream signaling pathways via its receptors. Specifically, through AdipoR1, adiponectin activates AMP-activated protein kinase (AMPK), while AdipoR2 mediates its effects through the MAPK and PPAR-α pathways. These interactions collectively influence steroid hormone gene expression by inhibiting the transcriptional activity of SP-1, ultimately regulating key reproductive processes such as testosterone synthesis and spermatogenesis [104,105]. Furthermore, exogenous adiponectin administration has been shown to increase the expression of StAR and 3β-HSD proteins in mouse testes, thereby promoting testosterone production [97]. However, it is important to note that this regulatory effect is not always stimulatory. For instance, Caminos et al. [92] observed that low to medium doses (10–100 ng/mL) of adiponectin inhibited StAR protein expression in rat testes and reduced testosterone synthesis. This highlights the dose-dependent nature of adiponectin’s effects, further underscored by findings in Simmental bulls, where a significant negative correlation exists between circulating testosterone levels and adiponectin concentrations [106].

In terms of sperm-related functions, adiponectin plays a key role in promoting spermatogenesis, sperm maturation, and motility. In rams, the mRNA expression levels of adiponectin and AdipoR1 correlate positively with sperm motility [107], and adiponectin has been shown to modulate sperm capacitation, impacting sperm structure and function [98]. In contrast, deficiency or ablation of AdipoR2 leads to seminiferous tubule atrophy and reduced sperm counts [108], a phenotype also linked to male infertility in humans [109]. Moreover, adiponectin exhibits significant protective effects on testicular health. In a high-fat diet-induced diabetic mouse model, recombinant globular adiponectin alleviated testicular injury by regulating autophagy, reducing endoplasmic reticulum stress, and mitigating oxidative stress. Conversely, adiponectin deficiency or marked reductions in adiponectin levels lead to reproductive dysfunction, as demonstrated by reduced testicular weight, decreased sperm counts, and impaired fertilization capacity in adiponectin-deficient mice [110]. Furthermore, reduced adiponectin levels in the testes of aged mice result in lower testosterone synthesis and compromised spermatogenesis [101], collectively emphasizing the crucial role of adiponectin in preserving testicular structural integrity, maintaining functional homeostasis, and delaying reproductive senescence in males.

## 6. Conclusions

Adiponectin, initially recognised for its role in metabolic regulation, is now increasingly understood to participate in the coordination of reproductive function through the HPG axis. By integrating evidence across the hypothalamus, pituitary, and gonads, this review highlights the significance of adiponectin as a metabolic signal linking energy homeostasis with reproductive endocrine regulation. By integrating evidence across the hypothalamus, pituitary, and gonads, this review highlights the significance of adiponectin as a metabolic signal linking energy homeostasis with reproductive endocrine regulation. Its receptors are widely distributed across this axis, providing a framework through which metabolic status can influence reproductive endocrine activity. Evidence from multiple species indicates that adiponectin acts at several levels of this system, including the regulation of GnRH secretion, the modulation of pituitary gonadotropin synthesis and release, and the control of gonadal gametogenesis and steroidogenesis. However, a coherent, axis-level view of adiponectin regulation remains incomplete. In particular, central mechanisms are still poorly defined, as the hypothalamic–pituitary unit has rarely been examined as a coordinated network. The direction and hierarchy of signalling interactions across levels, therefore, remain unclear. In addition, current research is largely centred on gonadal phenotypes, whereas the functional roles of adiponectin isoforms and their receptor-associated signalling pathways have not been systematically evaluated across tissues or physiological conditions. Interspecies variation further complicates interpretation. In birds, for example, the absence of a KISS1 orthologue suggests that adiponectin-dependent reproductive regulation may involve alternative neuroendocrine pathways that are not yet well characterised. On this basis, future progress is likely to come from three complementary directions. First, single cell and spatial omics should be deployed to establish a spatiotemporal atlas of adiponectin, AdipoR1 and AdipoR2, and downstream effectors across HPG cell types. Second, comparative and evolutionary genomics can be used to clarify which elements of adiponectin HPG coupling are conserved and which are lineage specific adaptations, providing a framework for interpreting discordant findings across models. Third, mechanism-informed intervention studies are needed to evaluate whether manipulation of adiponectin pathways can improve reproductive dysfunction associated with metabolic disorders. Future reviews may further address adiponectin isoform biology, tissue-specific receptor signaling, species-specific reproductive adaptations, and adiponectin-targeted strategies in metabolic disorder-associated reproductive dysfunction. Taken together, these efforts will help clarify how adiponectin links metabolic state to reproductive control and will support the development of more effective strategies for managing reproductive performance in animals.

## Data Availability

No new data were created or analyzed in this study. Data sharing is not applicable to this article.
